# Diffusion histology imaging differentiates distinct pediatric brain tumor histology

**DOI:** 10.1038/s41598-021-84252-3

**Published:** 2021-02-26

**Authors:** Zezhong Ye, Komal Srinivasa, Ashely Meyer, Peng Sun, Joshua Lin, Jeffrey D. Viox, Chunyu Song, Anthony T. Wu, Sheng-Kwei Song, Sonika Dahiya, Joshua B. Rubin

**Affiliations:** 1 Department of Radiology, Washington University School of Medicine, Room 3221, 4525 Scott Ave., St. Louis, MO 63110 USA; 2 Department of Pathology and Immunology, Washington University School of Medicine, 660 S. Euclid Avenue, St. Louis, MO 63110 USA; 3 Department of Pediatrics, St. Louis Children’s Hospital, Washington University School of Medicine, 660 S. Euclid Avenue, St. Louis, MO 63110 USA; 4 Department of Biomedical Engineering, Washington University, St. Louis, MO 63130 USA; 5 Department of Neuroscience, Washington University School of Medicine, St. Louis, MO 63110 USA; 6 Present Address: Keck School of Medicine, University of Southern California, Los Angeles, CA 9003389 USA; 7 Present Address: School of Medicine, University of Missouri – Kansas City, Kansas City, MO 64110 USA

**Keywords:** Cancer imaging, CNS cancer

## Abstract

High-grade pediatric brain tumors exhibit the highest cancer mortality rates in children. While conventional MRI has been widely adopted for examining pediatric high-grade brain tumors clinically, accurate neuroimaging detection and differentiation of tumor histopathology for improved diagnosis, surgical planning, and treatment evaluation, remains an unmet need in their clinical management. We employed a novel Diffusion Histology Imaging (DHI) approach employing diffusion basis spectrum imaging (DBSI) derived metrics as the input classifiers for deep neural network analysis. DHI aims to detect, differentiate, and quantify heterogeneous areas in pediatric high-grade brain tumors, which include normal white matter (WM), densely cellular tumor, less densely cellular tumor, infiltrating edge, necrosis, and hemorrhage. Distinct diffusion metric combination would thus indicate the unique distributions of each distinct tumor histology features. DHI, by incorporating DBSI metrics and the deep neural network algorithm, classified pediatric tumor histology with an overall accuracy of 85.8%. Receiver operating analysis (ROC) analysis suggested DHI’s great capability in distinguishing individual tumor histology with AUC values (95% CI) of 0.984 (0.982–0.986), 0.960 (0.956–0.963), 0.991 (0.990–0.993), 0.950 (0.944–0.956), 0.977 (0.973–0.981) and 0.976 (0.972–0.979) for normal WM, densely cellular tumor, less densely cellular tumor, infiltrating edge, necrosis and hemorrhage, respectively. Our results suggest that DBSI-DNN, or DHI, accurately characterized and classified multiple tumor histologic features in pediatric high-grade brain tumors. If these results could be further validated in patients, the novel DHI might emerge as a favorable alternative to the current neuroimaging techniques to better guide biopsy and resection as well as monitor therapeutic response in patients with high-grade brain tumors.

## Introduction

Pediatric brain tumors are the second most common childhood malignancy and the most common solid tumor in children^1^. Pediatric brain cancer has unfortunately surpassed leukemia to become the most common cause of childhood cancer death in the US^2^. It is estimated that 2940 new cases of childhood (0–14 age group) and adolescent (15–19 age group) primary malignant and non-malignant central nervous system (CNS) tumors will be diagnosed in the United States in 2020^3^.

In the past two decades, the advent of neuroimaging technologies has enabled clinicians to detect tumor recurrence or dissemination with improved certainty^4^. However, these discoveries were developed largely for adult brain tumor patients, which are most often biologically distinct from those that occur in children, which exhibit unique genomic and imaging characteristics^5^. Evaluating pediatric brain tumors is often a diagnostic challenge due to their diverse tumor pathologies, nonspecific or overlapping imaging findings, susceptibility artifacts from intratumoral calcification or hemorrhage, and motion artifacts in young children^6^. Conventional MRI-based diagnoses also fail to offer adequate information regarding the specific tumor type, tumor grade, tumor viability, and treatment response of lesions. Although advanced MRI techniques like diffusion tensor imaging (DTI), perfusion MRI, MR spectroscopy (MRS), and susceptibility-weighted imaging (SWI) are incorporated into clinical MRI protocols, they still fall short^6–8^. The widely used multiparametric magnetic resonance imaging (mpMRI) approach fails to accurately reflect tumor histopathology such as tumor cellular density, necrosis, hemorrhage, or infiltrative edges. As prior studies documented the histological and radiological tumor heterogeneity coexisting within high grade tumor lesions, it is imperative to develop a technique capable of discerning the varied appearance of these lesions non-invasively^9^.

We have previously developed diffusion basis spectrum imaging (DBSI)^10^ demonstrating its ability to quantitatively characterize pathologies in multiple central nervous system diseases, including, multiple sclerosis^11–14^, spinal cord injury^15^, and epilepsy^16^. More recently, we developed a novel diffusion histology imaging (DHI) approach that incorporates DBSI-derived diffusion metrics as the input for a deep neural network (DNN) algorithm to detect and differentiate underlying pathologies in pediatric high-grade brain tumors.

## Materials and methods

### Study design

This study has been approved by the institutional review board of Washington University School of Medicine. Informed consent was obtained from all subjects’ parents and/or legal guardians for the use of samples in this study. All methods were carried out in accordance with relevant guidelines and regulations. Nine post-mortem pediatric brain tumor specimens that were part of the Washington University Legacy Project were included for the study. Among these nine pediatric patients, four were male and five were female.

### Postmortem brain specimen

**Figure 1:**
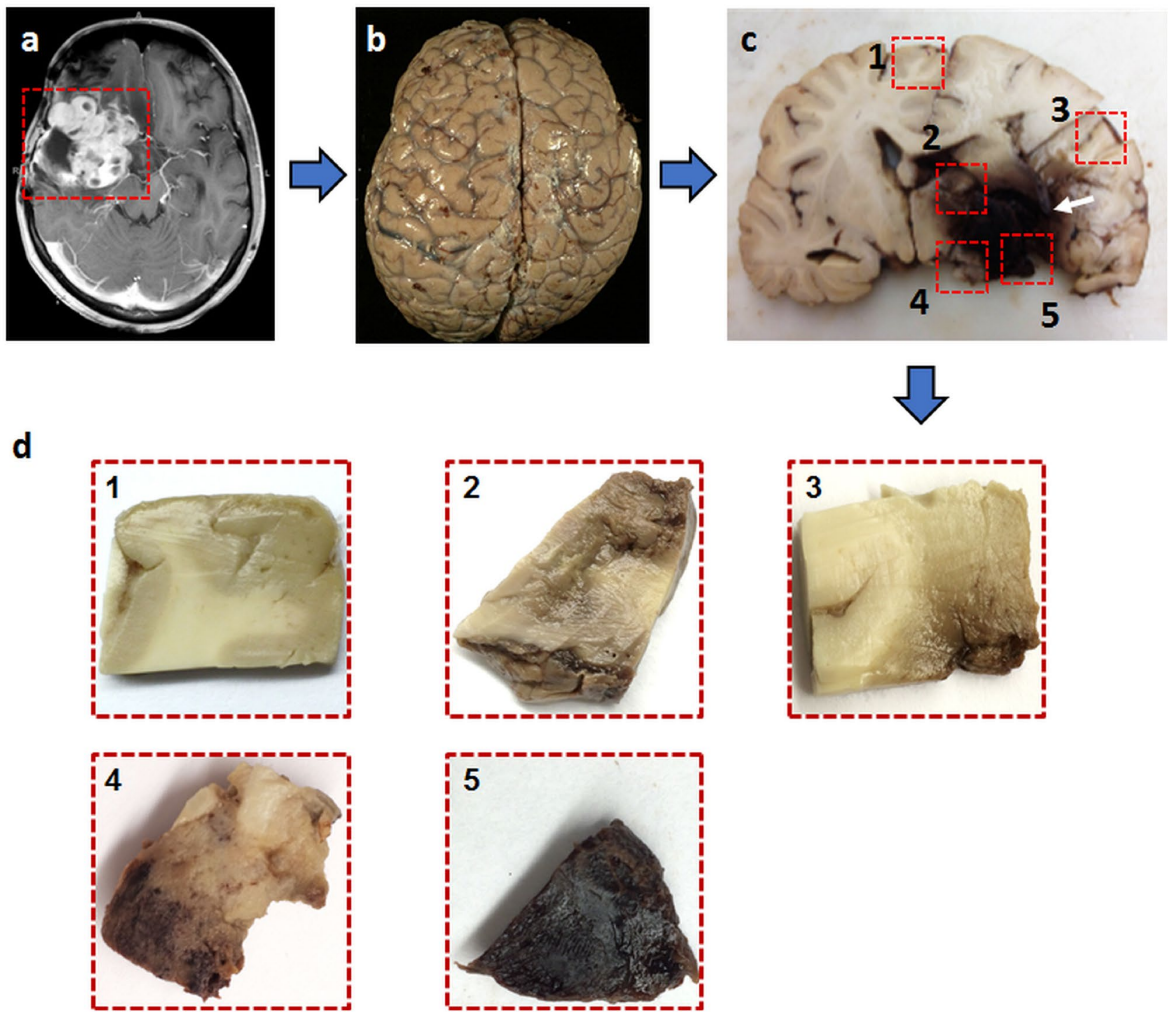
Illustration of brain specimen procurement from a patient with high-grade pediatric brain tumor. (**a**) In vivo Gd-enhanced T1-weighted image indicated a large lesion (square) with heterogeneous intensities in the right posterior region from a 16-year-old patient with embryonal neoplasm (WHO Grade IV). (**b**) Brain specimen was procured and immediately formalin-fixed. (**c**) Coronal slices revealed a large tumor with admixed hemorrhage and necrosis in the right thalamus (arrow). (**d**) Five tissue blocks were prepared in total i.e. from tumor (block 2, blcok 4), tumor interface with normal adjacent brain (block 3), hemorrhage and necrosis (block 5), as well as grossly normal brain (block 1) (**c**).

The autopsy was performed within 24 h of the death of the patients to prevent the deterioration of the tissue. The brain specimen was immediately fixed in formalin right after the autopsy (Fig. 1b). The tissue blocks were obtained from the brain specimen no sooner than seven days after the formalin fixation (Fig. 1c). A total of 45 samples were resected from tumor, tumor interface with normal adjacent brain, areas of hemorrhage and necrosis, as well as normal brain tissue (Fig. 1c). The average size of the specimens was 21 mm ± 4 mm.

### Ex vivo MRI of brain specimen

Brain tumor specimens were submersed in formalin for ex vivo imaging to keep tissue from dehydration during study. The specimens were examined using a 4.7-T Agilent/Varian MR scanner (Agilent Technologies, Santa Clara, CA) and a custom-built circular surface coil (3.5-cm diameter). A multi-echo spin-echo diffusion weighted sequence using 99 diffusion-encoding directions with maximum b-values = 3000 s/mm^2^ was employed to acquire diffusion-weighted images. The imaging parameters were as follows: repetition time (TR) = 1500 ms, echo time (TE) = 40 ms, time between application of gradient pulse 20 ms, diffusion gradient on time 8 ms, slice thickness 0.5 mm, field-of-view (FOV) 32 × 32 mm^2^, data matrix 128 × 128, number of average 1, in-plane resolution 0.25 × 0.25 mm^2^. T2W images were acquired with a multi-slice spin echo sequence with TR = 4000 ms, echo time TE = 80 ms, FOV 32 × 32 mm^2^, data matrix 128 × 128. T1W images were acquired with a gradient echo sequence with TR = 80 ms, TE = 10 ms, FOV 32 × 32 mm^2^, data matrix 128 × 128, 8 averages.

### DBSI analysis of brain tumor

DBSI models brain tumor diffusion-weighted MRI signals as a linear combination of discrete multiple anisotropic diffusion tensors and a spectrum of isotropic diffusion tensors:

$$ \frac{{S_{k} }}{{S_{0} }} = \mathop \sum \limits_{i = 1}^{{N_{Aniso} }} f_{i} e^{{ - \left| {\overrightarrow {{b_{k} }} } \right|\lambda_{ \bot i} }} e^{{ - \left| {\overrightarrow {{b_{k} }} } \right|\left( {\lambda_{\parallel i} - \lambda_{ \bot i} } \right)\cos^{2} \phi_{ik} }} + \mathop \smallint \limits_{a}^{b} f\left( D \right)e^{{ - \left| {\overrightarrow {{b_{k} }} } \right|D}} dD\quad \left( {k = 1,2,3, \ldots } \right) $$

In^1^, *b*_*k*_ is the *k*th diffusion gradient; *S*_*k*_/*S*_*0*_ is the acquired diffusion-weighted signal at direction of *b*_*k*_ normalized to non-diffusion-weighted signal; *N*_*Aniso*_ is number of anisotropic tensors to be determined; *ϕ*_*ik*_ is the angle between diffusion gradient (*b*_*k*_) and principal direction of the *ith* anisotropic tensor*;*$\left| {\overrightarrow {{b_{k} }} } \right|$ is *b*-value of the *kth* diffusion gradient; *λ*_*||i*_ and *λ*_⟂*i*_ are axial and radial diffusivity of the *ith* anisotropic tensor under the assumption of cylindrical symmetry; *f*_*i*_ is signal-intensity-fraction of the *ith* anisotropic tensor; *a, b* are low and high diffusivity limits of isotropic diffusion spectrum; *f*(*D*) is signal-intensity-fraction at isotropic diffusivity *D*.

Based on our ex vivo MRI and histological analyses of resected specimens from previous studies^17^, the following isotropic-diffusion profiles were established. We observed that highly restricted isotropic diffusion (0 ≤ D ≤ 0.2 μm^2^/ms) is associated with lymphocytes; restricted-isotropic diffusion (0.2 < D ≤ 0.8 μm^2^/ms) is associated with dense tumor cellularity; and hindered-isotropic diffusion (0.8 < D ≤ 2 µm^2^/ms) is associated with tumor necrosis. DBSI provides a simple tensor expression for individual image voxels to visualize morphological features secondary to tumor formation, some of which are not as discretely detectable by conventional MRI. It is the sensitivity of diffusion-weighted MRI signal to the microstructural changes that allows DBSI to more precisely reflect morphological changes resulting from tumor presence or other pathologic alterations. By using this feature of DBSI as the input for deep neural network algorithms, we created DHI to recapitulate histopathologic analysis using MRI.

### Histologic staining and evaluation

The formalin-fixed tissue was embedded in paraffin after scanning. The paraffin embedded tissue was then sequentially sectioned at 5-μm thickness and stained with hematoxylin and eosin (H&E). Histology slides were digitized using NanoZoomer 2.0-HT System (Hamamatsu, Japan) with a 20 × objectives for analyses. Two experienced neuropathologists (K.S. and S.D.) reviewed all the histological slides with a consensus on the selected tumor histopathologic features. Regions of normal white matter (WM), densely cellular tumor (DC tumor), less densely cellular tumor (LDC tumor), necrosis, tumor infiltrative edge, and hemorrhage were outlined and drawn on H&E images with 20× magnification.

### Image processing

Voxel-wise DTI and DBSI analyses were performed by an in-house software developed using MATLAB® (MathWorks; Natick, MA). The computation time for DTI and DBSI for each specimen is about 40 min.

### Co-registration between histology images and ex vivo MRI

**Figure 2:**
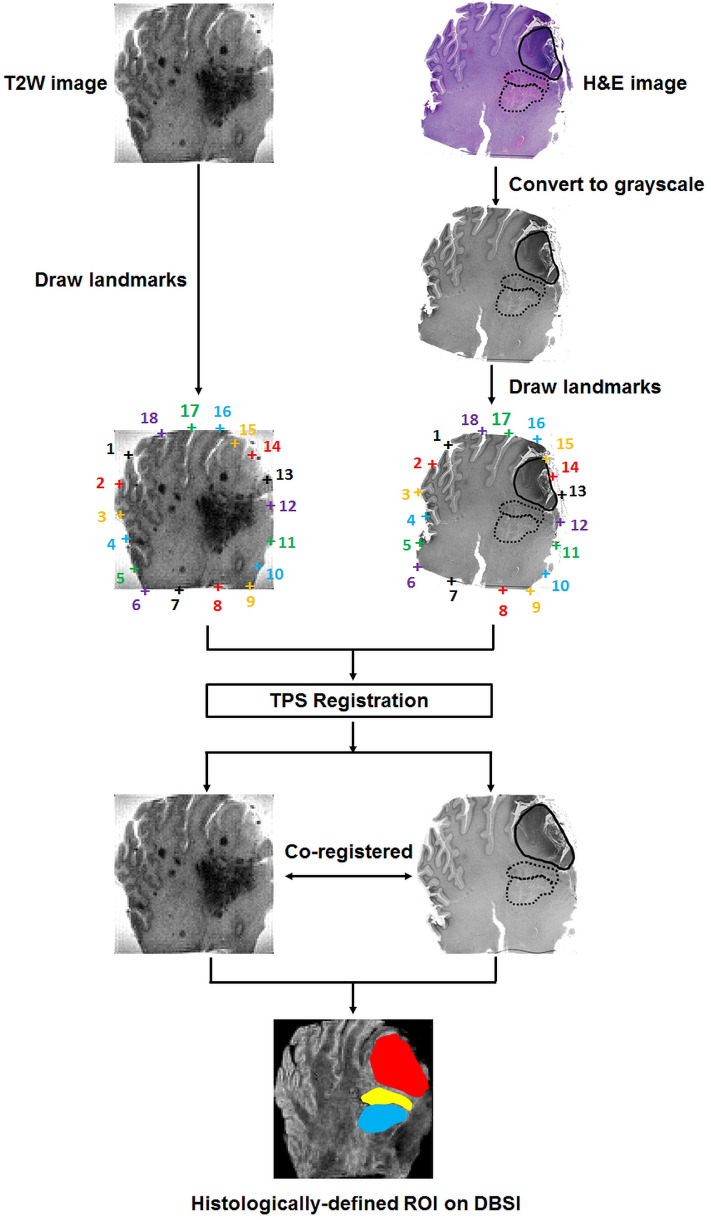
Co-registration between histology and MRI. Raw RGB H&E images were first converted to grayscale images for enhanced coregistration. Eighteen pairs of landmarks along the perimeter of the brain specimens were manually placed on the MR image and grayscale H&E image. The transformation matrix of the two-dimensional thin plate spline (TPS) registration was computed in *MIPAV* (version 10.0.0) and applied to warp the H&E image to the orientation of the MR image. After co-registration, the pathologist defined tumor histology regions on H&E images. These were then successfully transferred to the corresponding MR image.

The two dimensional (2D) thin plate spline (TPS) registration was performed using *MIPAV* (Version 10.0.0, NIH; https://mipav.cit.nih.gov/index.php) as described in our previous study^17^ to co-register the histology images with MR images. To achieve successful co-registration, we first ensured the plane of histology section of the brain tumor specimens matched closely with the slice plane of the corresponding T2-weighted (T2W) images. For the co-registration preprocessing, the RGB format of H&E images with histology annotations were converted to grayscale format to match with T2W images (Fig. 2) using the Pillow package in Python 3.6.8 (https://pillow.readthedocs.io/en/3.1.x/index.html#). Afterwards, eighteen pairs of landmarks along the perimeter of each specimen were manually placed on both H&E images and T2W images (inherently co-registered with DTI and DBSI maps) to compute the transformation matrix for matching H&E images with MRI (Fig. 2). Through successful image co-registration, the regions of interest (ROI) with pathologist-identified tumor histology on the H&E images can be transferred to DBSI for further analysis (Fig. 2).

### Deep neural network (DNN) model development and optimization

Our complete dataset consisted of 99,388 imaging voxels from 45 specimens obtained from 9 patients. The collected voxels were split into training, validation, and test datasets with an 8:1:1 ratio, respectively. Imaging voxels from test datasets were separated and distinct from the ones that were used in the training and validation steps. Validation set was employed to fine tune the model hyper-parameters. To balance data from groups of different tumor histologic components, a synthetic minority oversampling technique (SMOTE)^18^ was applied to over-sample the minority group by introducing synthetic feature samples. This data balancing approach has been demonstrated to be beneficial for avoiding over-fitting and improving model generalization^18,19^. Data balancing were only applied to the training dataset, while the validation and test dataset was kept unchanged. The diffusion metrics assessed with our DNN modeling included 10 diffusion metrics provided from DBSI. Specifically, DBSI metrics include mean apparent diffusion coefficient (ADC), mean FA, fiber fraction, fiber fractional anisotropy (FA), fiber axial diffusivity (AD), fiber radial diffusivity (RD), restricted isotropic diffusion fraction (restricted fraction), restricted isotropic diffusivity, hindered isotropic diffusion fraction (hindered fraction), hindered isotropic diffusivity, free isotropic diffusion fraction (free fraction), free isotropic diffusivity.

A supervised deep neural network (DNN) was adopted to detect and classify tumor histologic components by referencing the H&E findings. The DNN model was developed using TensorFlow 2.0 framework in Python 3.6.8^20^. In general, the DNN model was equipped with ten fully connected hidden layers. Batch normalization layer with a mini-batch size of 200 was used before feeding data to the next hidden layer to improve model optimization and prevent overfitting. Exponential linear units^21^ were adopted to activate specific functions in each hidden layer. The final layer was a fully connected softmax layer that generated a likelihood distribution of six output classes. We used Adam optimizer with the default parameters of β_1_ = 0.9, β_2_ = 0.999 and mini-batch size of 200. The learning rate was manually tuned to achieve the fastest convergence. We chose cross-entropy as the loss function and trained the model to minimize the error rate on the validation dataset. Overall, hyper-parameters for the DNN architecture and optimization algorithm were chosen through a combination of grid search and manual tuning.

### Statistical analysis

Statistical differences in diffusion metrics between the tumor histology groups were evaluated by the Mann–Whitney U test. The results were presented as mean ± standard deviation. A *p* < 0.05 was considered statistically significant. In multi-class classification, confusion matrices were calculated and used to illustrate the specific examples of tumor histologic components where the model prediction agrees with the pathologists’ diagnoses. We also used one-versus-rest strategy to perform receiver operating characteristics (ROC) analysis. Area under curve (AUC) was calculated to assess model discrimination of each tumor histological component. Sensitivity and specificity values were calculated using Youden Index^22^. The precision-recall curve and F_1_-scores were also calculated to provides complementary information to the ROC curves. F_1_-score (ranges from 0 to 1) favors models that maximize both precision and recall simultaneously, which is especially helpful to address the insensitivity of AUC on class imbalance. The 95% confidence interval values were calculated using the percentile bootstrap method with 10,000 independent experiments^23^. All the statistical metrics and curves were calculated using the SciPy^24^ and Scikit-learn^25^ packages with Python version 3.6.

## Results

### A brief description of tumor specimens examined

Postmortem brain tumor specimens examined were from 9 pediatric brain tumor patients, aged from 4 to 17 years old at the time of initial diagnosis. The mean age was 10.8 ± 3.7 years old. The patients' age at autopsy ranged from 7 to 18, with a mean of 13.1 ± 3.7 years. Four tumors were located in the brainstem, two in the thalami, one in the right cerebral cortex, and one at the cerebellopontine angle. These were confirmed to be diffuse midline gliomas with H3K27M mutation by immunohistochemistry (n = 4), glioblastoma (n = 3), and embryonal tumor with multilayered rosettes with LIN28A protein overexpression (medulloepithelioma phenotype, NEC; n = 1). One patient with neurofibromatosis 1 (NF1) had three different tumors at three distinct time points; these were an optic pathway glioma (pilocytic astrocytoma), a diffuse astrocytoma, WHO grade II involving the right parieto-temporal lobe, and a CNS embryonal tumor involving the right temporal lobe. All details are summarized in Table 1.

**Table 1:** Patient information.

Patient ID	Age at diagnosis	Age at post-mortem	Gender	Location	Histologic diagnosis	Molecular alterations
WU-1	9	9	F	Thalamus	Diffuse midline glioma, WHO grade IV	H3K 27M mutant (by immunohistochemistry
WU-2	11	14	M	Left temporal lobe	Glioblastoma, IDH wildtype, WHO grade IV	Tumor progressed from IDH wildtype anaplastic astrocytoma. Next generation sequencing showed *CREBBP* G1479 alteration
WU-3	11	12	F	Right parietal-occipital lobe	Diffuse midline glioma, WHO grade IV	H3K 27M (by immunohistochemistry
WU-4	7	16	M	1. Right temporal lobe	CNS embryonal tumor with anaplastic features, WHO grade IV (2013)	Background of NF1 with three tumors at different time points
				2. Right posterior temporoparietal lobe	Diffuse astrocytoma, WHO grade II (2006)	18 non-synonymous variants were identified by next generation sequencing *TP53*, p.R213Dfs*34, *TP53* and p.T211I
				3. Optic pathway	Pilocytic astrocytoma, WHO grade I (not sampled until post-mortem)	*MAP2K2*,p.I369V
WU-5	10	10	M	Pons	Diffuse midline glioma, WHO grade IV	H3K 27M mutant (by immunohistochemistry
WU-6	13	14	M	Pons	Diffuse midline glioma, WHO grade IV	H3K 27M mutant (by immunohistochemistry
WU-7	4	7	F	Right cerebellopontine angle	Embryonal tumor with multilayered rosettes, medulloepithelioma phenotype, WHO grade IV, NEC	FISH could not demonstrate C19MC alteration but multifocal LIN28A protein expression was seen by immunohistochemistry
WU-8	17	18	M	Right cerebral hemisphere (extensive involvement left side, brainstem and cerebellum)	Glioblastoma, IDH wildtype, WHO grade IV	Loss of 10q (*PTEN*) and monosomy 10 (by FISH); no *EGFR* amplification or polysomy of chromosome 7
WU-9	15	18	F	Epicenter in brainstem, right thalamus, right basal ganglia, and cerebellum	Glioblastoma, IDH wildtype, WHO grade IV	H3K 27M negative (by immunohistochemistry)

### Relating MRI metrics to tumor pathologies

**Figure 3:**
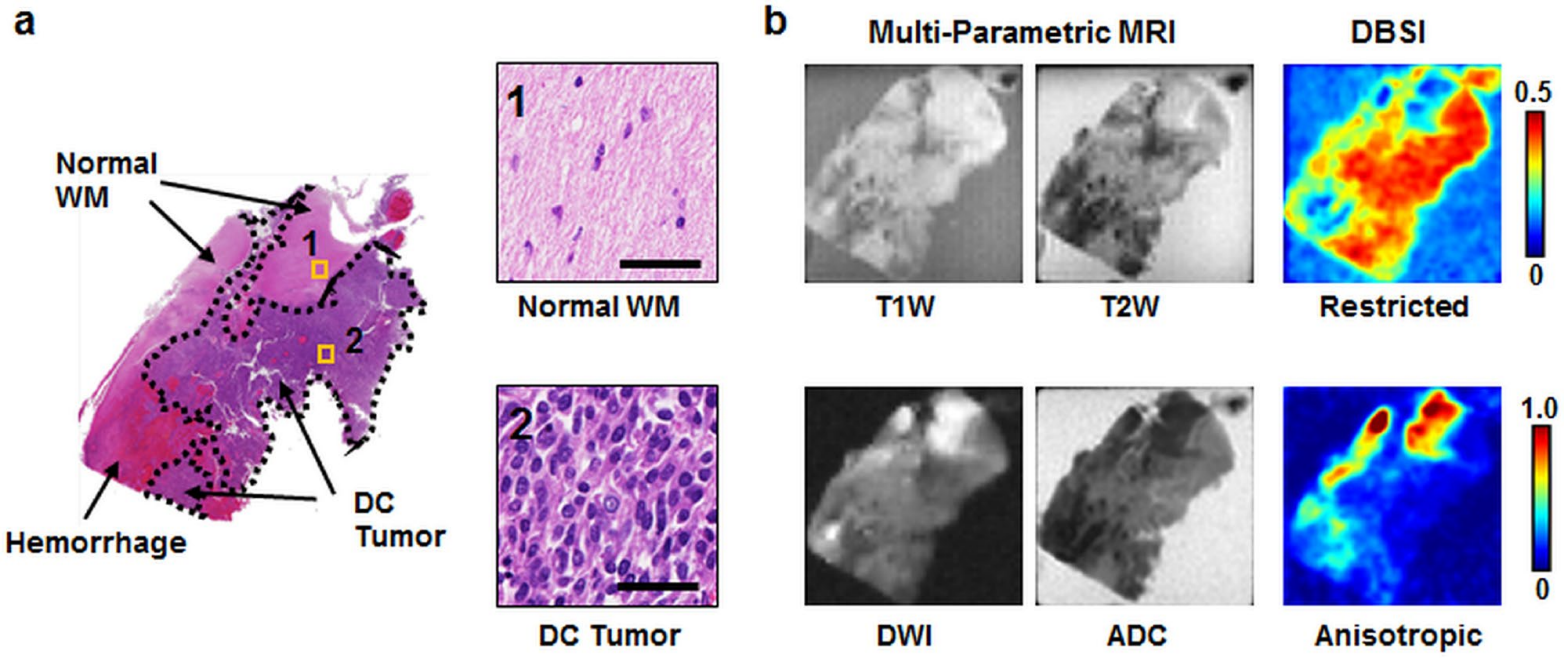
A representative tissue block was imaged with ex vivo MRI, followed by histologic processing and evaluation. (**a**) H&E image of a sectioned specimen after ex vivo MRI with regions of white matter, densely cellular tumor and hemorrhage outlined for assessing the efficacy of metrics derived by multi-parametric MRI and DBSI. The expanded region of densely cellular tumor features characteristically increased cellularity. Scale bar measures 50 um. (**b**) T1W and T2W MRI did not distinguish densely cellular tumor region from white matter or hemorrhage. Most strikingly, diffusely cellular tumor region exhibited lower DWI and higher ADC countering the conventional wisdom that higher tumor cellularity is associated with restricted diffusion. *WM* white matter, *DC* tumor densely cellular tumor.

Figures 1 and 3 show a representative case from a 16-year-old brain tumor patient with embryonal neoplasm (WHO Grade IV). Clinical gadolinium (Gd)-enhanced T1-weighted imaging of this patient several weeks prior to death revealed a new lesion in the right temporal lobe of the brain (Fig. 1a, square). At autopsy, the brain was removed and immediately suspended in formalin for fixation (Fig. 1b). Coronal slices exhibited a large hemorrhagic and necrotic tumor mass with its epicenter in the right thalamus (Fig. 1c). Tissue blocks were obtained from this region (Fig. 1d) for ex vivo imaging (Fig. 2). Of note, this patient had two other known tumors, one in his optic pathway (WHO grade I) and another diffuse astrocytoma (WHO grade II) in right posterior temporo-parietal lobe. The boundaries of latter were however relatively indistinct from the high-grade hemorrhagic and necrotic embryonal neoplasm (WHO grade IV) by gross examination alone.

A densely cellular tumor region and white matter (WM) were indistinguishable in both T1-weighted image (T1WI) and T2-weighted image (T2WI) (Fig. 3). Hemorrhage appeared hypointense compared to other regions in T1WI and T2WI (Fig. 3). White matter and the hemorrhagic region appeared hyperintense in diffusion-weighted image (DWI) and hypointense in ADC map comparing with the densely cellular tumor regions. Densely cellular tumor appeared to associate with high restricted fraction.

### DBSI derived diffusion metrics reflected tumor pathologies

**Figure 4:**
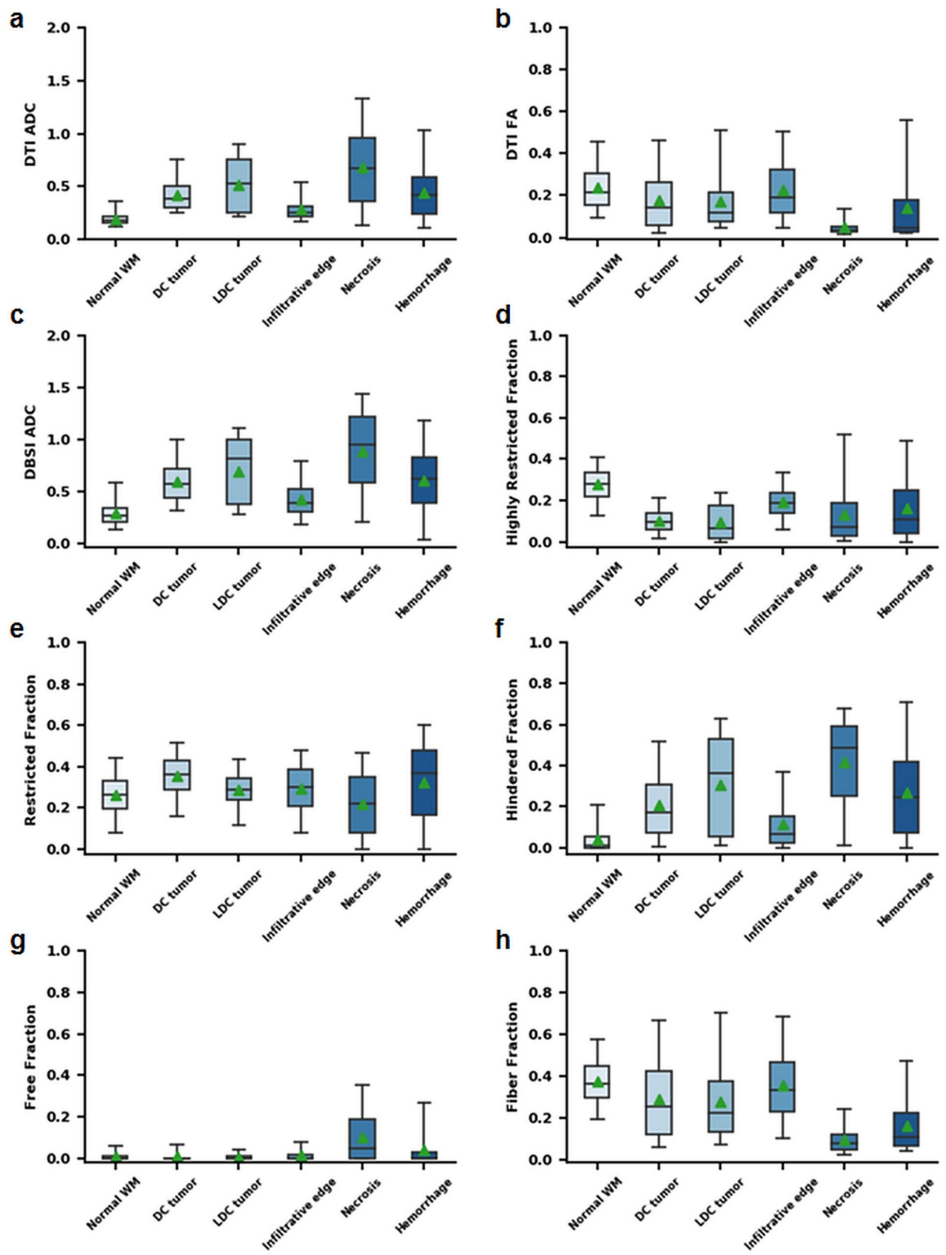
Group analysis on different tumor histologic components on representative diffusion metrics including (**a**) ADC, (**b**) DTI FA, (**c**) DBSI isotropic ADC, (**d**) highly restricted fraction, (**e**) restricted fraction, (**f**) hindered fraction, (**g**) free fraction, and (**h**) fiber fraction. Particularly, normal WM and the infiltrative edge showed higher fiber fraction and DTI-FA than the other tumor histologies. DC tumor and LDC tumor showed higher restricted fraction values than other histologies. Necrosis showed higher ADC, hindered fraction and free fraction values as well as lower restricted fraction, fiber fraction and DTI-FA compared to the other histologies. These findings were collectively consistent with DBSI’s modelling for malignant brain tumor. ADC, µm^2^/ms. *Normal WM* normal white matter, *DC tumor* densely cellular tumor, *LDC tumor* less densely cellular tumor.

After MR images were co-registered with H&E images, image-voxels from segmentations of the five different tumor pathologies were subsequently obtained and plotted for group comparison (Fig. 4). The ADC of densely cellular tumor (0.43 ± 0.17 µm^2^/ms) was 115% higher (*p* < 0.05) than then normal white matter and 42% higher (*p* < 0.05) than the infiltrative edge (0.30 ± 0.15 µm^2^/ms). Additionally, the densely cellular tumor was 17% lower (*p* < 0.05) than the less densely cellular tumor (0.52 ± 0.26 µm^2^/ms), and 37% lower (*p* < 0.05) than the necrosis (0.68 ± 0.37 µm^2^/ms) (Fig. 4a). DTI fractional anisotropy (FA) value of the tumor infiltrative edge was similar to that of white matter (0.23 ± 0.14 vs. 0.24 ± 0.11) (Fig. 4b). The comparison of isotropic ADC values derived by DBSI among tumor pathologies revealed a similar trend as seen in mean ADC and were consistently higher than mean ADC (Fig. 4c). For the highly restricted fraction, WM exhibited much higher values than other pathologies. Densely and less densely cellular tumors exhibited the least highly restricted fraction values among all histologic features (Fig. 4d). For the restricted fraction, densely cellular tumor (0.35 ± 0.10) showed 35%, 21%, 21% and 59% higher (all *p* < 0.05) values than normal WM (0.26 ± 0.11), less densely cellular tumor (0.29 ± 0.09), infiltrative edges (0.29 ± 0.12) and necrosis (0.22 ± 0.15), respectively. This result correlated well with the expected cellularity decrease from densely cellular, less densely cellular tumor regions to necrotic tissue (Fig. 4e). As expected, necrosis was characterized by higher hindered fraction (0.42 ± 0.21) and free fraction values (0.11 ± 0.12) than any of the other histologic features. In the anisotropic fraction, normal WM (0.38 ± 0.12) and infiltrative edge (0.36 ± 0.17) had similar values; this anisotropic component was much higher than other histologic components.

### Classifications of tumor histologic components

**Figure 5:**
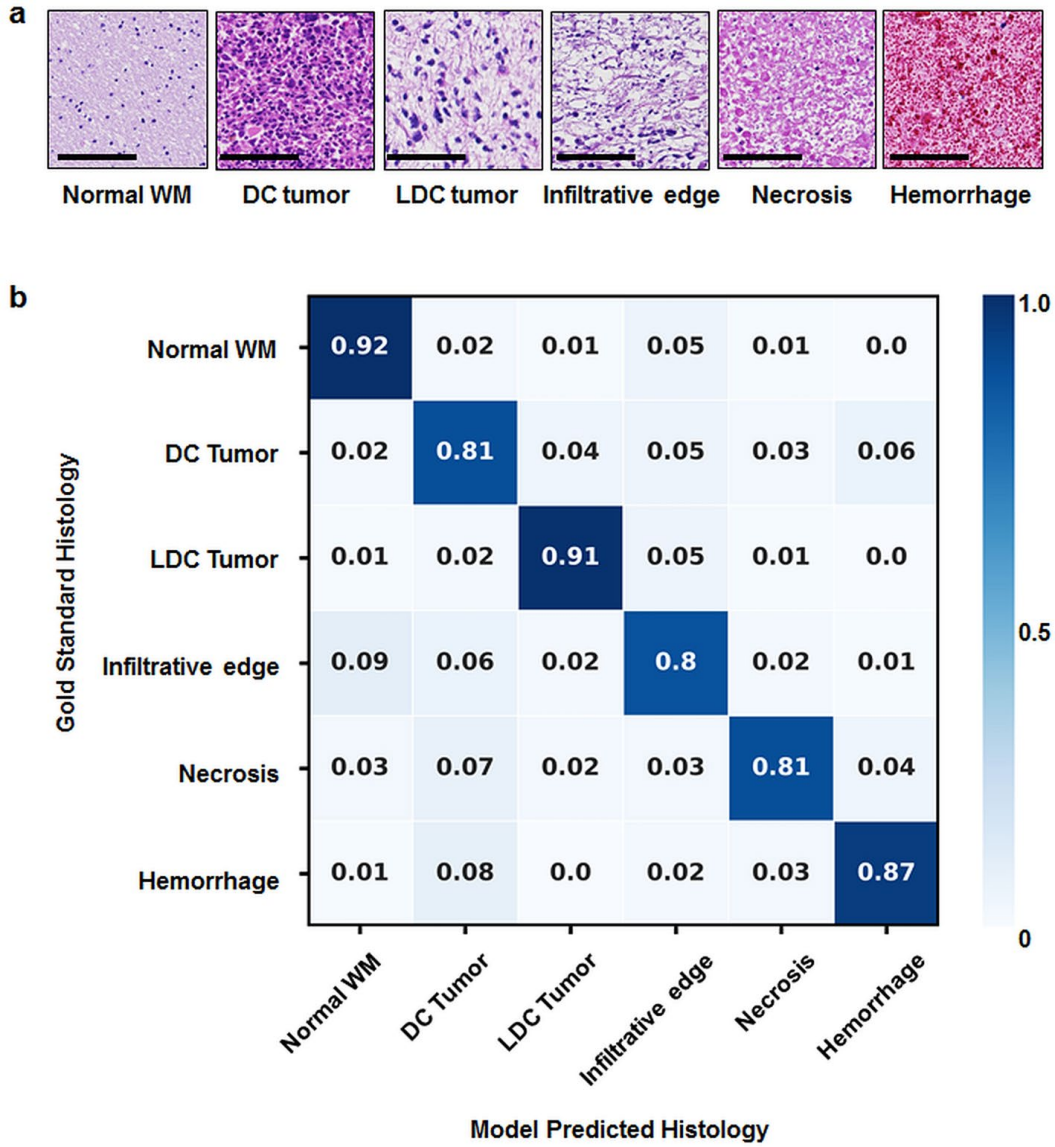
(**a**) Representative H&E images of normal white matter, densely cellular tumor, less densely cellular tumor, infiltrative edge, necrosis and hemorrhage, respectively. (**b**) Independent test dataset confusion matrix for the predictions of DHI versus gold standard, i.e. histologic examination (n = 9939). Rows contain tumor histologic classifications identified by a neuropathologist, and columns represent tumor histologic classifications as predicted by DHI. Scale bar measures 100 μm. *Normal WM* normal white matter, *DC tumor* densely cellular tumor, *LDC tumor* less densely cellular tumor.

It was clear that DBSI is more specific to tumor pathologies than conventional MRI metrics. To alleviate overlap between DBSI-derived metrics and tumor pathologies, we sought to classify tumor pathologies using DHI, by combining DBSI-derived structural metrics with DNN algorithm. We first performed a multi-class classification of normal WM, densely cellular tumor, less densely cellular tumor, infiltrative edge, necrosis, and hemorrhage regions containing a total of 143,100 image voxels to train the DHI model after oversampling for data balancing. Representative H&E images corresponding to one MRI voxel revealed distinct histologic features (Fig. 5a). For the independent test set (n = 9939), we achieved an overall accuracy of 85.8%. Confusion matrix analysis indicated strong concordance between DHI predictions and the neuropathologist-identified pathological features (Fig. 5b). DHI accurately predicted normal WM, densely cellular tumor, less densely cellular tumor, infiltrative edge, necrosis, and hemorrhage, with true positive rates of 91.6%, 80.6%, 90.8%, 80.2%, 81.4% and 86.7%, respectively.

**Figure 6:**
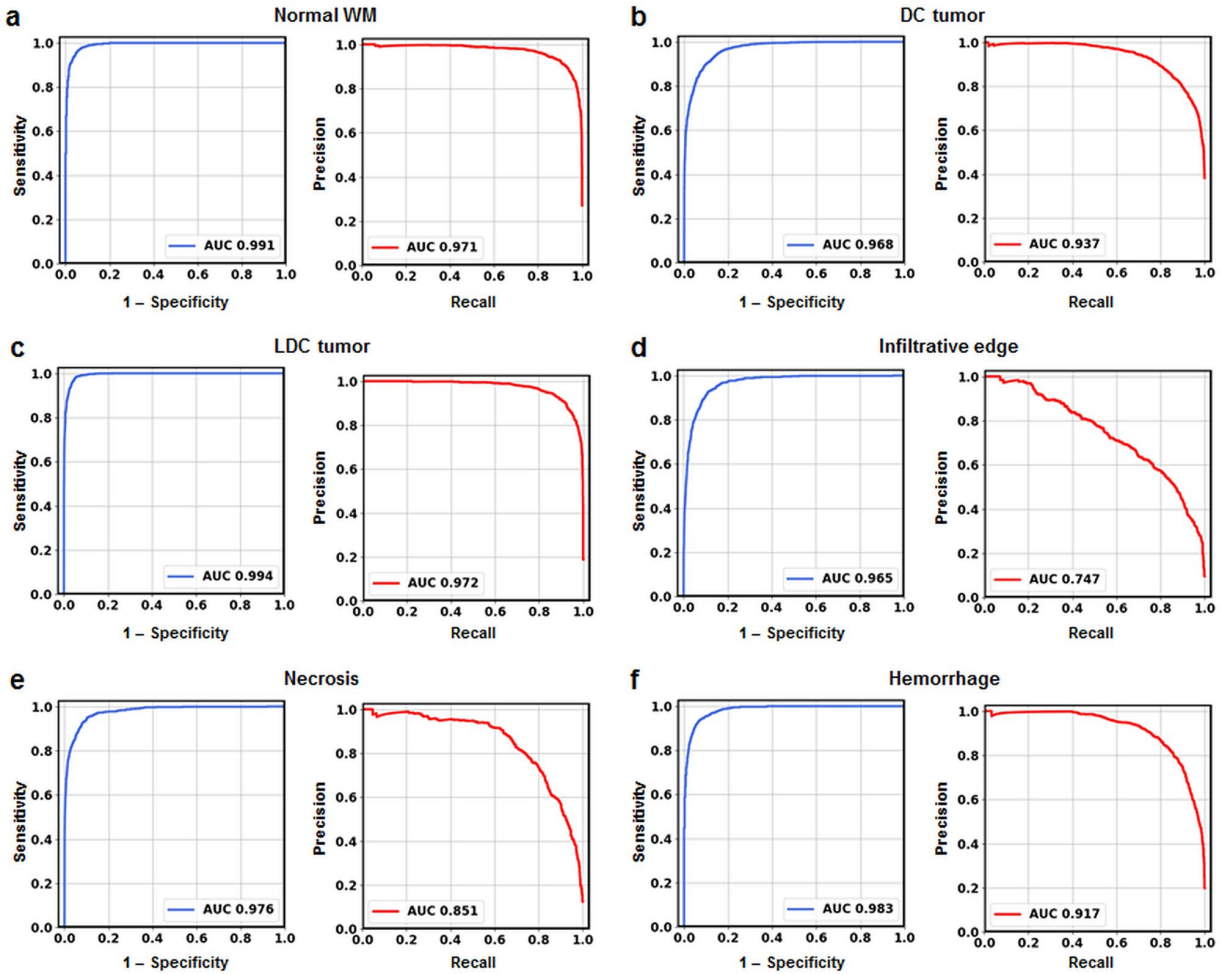
Receiver operating characteristics (ROC) curves and precision-recall (PR) curves calculated using one-vs-rest strategy for 6 different tumor histological components including (**a**) normal white matter, (**b**) densely cellular tumor, (**c**) less densely cellular tumor, (**d**) tumor infiltrative edge, (**e**) tumor necrosis and (**f**) hemorrhage. All 6 ROC curves showed high areas under curve (AUC), indicating strong sensitivity and specificity in detecting these tumor histologic components. Tumor infiltrative edge did not perform as well as other histologic components in precision-recall analysis, indicating that tumor infiltration could be overestimated by the model. *Normal WM* normal white matter, *DC tumor* densely cellular tumor, *LDC tumor* less densely cellular tumor.

To test DHI’s ability to distinguish each individual tumor pathology, we adopted a one-versus-rest strategy to perform receiver operating characteristic (ROC) and precision-recall analysis (Fig. 6). The ROC curves indicated high area under curve (AUC) values in the differentiation of all six different histologic components (Fig. 6a–f). We calculated 95% confidence intervals (CI) of AUCs using the percentile bootstrap method with 10,000 iterations. The AUC values (95% CI) were 0.984 (0.982–0.986), 0.960 (0.956–0.963), 0.991 (0.990–0.993), 0.950 (0.944–0.956), 0.977 (0.973–0.981) and 0.976 (0.972–0.979) for normal WM, densely cellular tumor, less densely cellular tumor, infiltrative edge, necrosis and hemorrhage, respectively (Table 2). We also calculated sensitivity and specificity for each class under the Youden Index. All the sensitivity values were higher than 91% with specificity values higher than 85% (Table 2). We also calculated precision-recall curves and F_1_-scores to provide complementary information to address ROC analyses’ insensitivity to class imbalance and the possible overestimation of model performance. The precision-recall curves performed inferiorly on tumor infiltration (Fig. 6d, AUC 0.747) and necrosis (Fig. 6e, AUC 0.851) when compared to other tumor histologic regions. Similarly, the F_1_-scores of the infiltrative edge (0.698) and necrosis (0.799) were worse than those of normal white matter (0.918), densely cellular tumor (0.850), less densely cellular tumor (0.911), and hemorrhage (0.848) (Table 2).

**Table 2:** Diagnostic performances of DHI in classifying different tumor histologies.

Tumor histology	Sensitivity (%)	Specificity (%)	AUC (95% CI)	F_1_-score
Normal WM	94.1 (92.8–97.4)	93.3 (89.6–94.3)	0.984 (0.982–0.986)	0.918
DC tumor	88.9 (87.2–90.6)	89.8 (88.2–91.3)	0.960 (0.956–0.963)	0.850
LDC tumor	97.3 (96.3–98.1)	93.8 (92.9–94.7)	0.991 (0.990–0.993)	0.911
Infiltrative edge	90.8 (87.6– 94.2)	86.7 (83.2–89.1)	0.950 (0.944–0.956)	0.698
Necrosis	94.5 (92.4–96.8)	90.0 (87.7–91.6)	0.977 (0.973–0.981)	0.799
Hemorrhage	91.5 (88.8–94.4)	92.2 (89.2–94.5)	0.976 (0.972–0.979)	0.848

The 95% confidence interval (CI) values were calculated using percentile bootstrap method with 10,000 independent experiments.

*CI* confidence interval, *Normal WM* normal white matter, *DC tumor* densely cellular tumor, *LDC tumor* less densely cellular tumor.

## Discussion

Pediatric brain tumors are the leading cause of cancer-related death in children. Current curative approaches in management rely, in most cases, on complete surgical resection, followed by irradiation and chemotherapy^4,26^. Histologic assessment of tumor cellularity, infiltration and necrosis is critical in the diagnosis and grading, as well as subsequent clinical decision-making for patient management and follow-up^27^. The current clinical gold standard, i.e. histologic examination, requires stereotactic biopsy or surgical resection^28^, which carries potential risks including infections, seizures, stroke, coma, as well as brain swelling or bleeding^29^. Sometimes inconclusive pathological findings result from inadequate sampling, necessitating repeat biopsy, with all its attendant risks^30^. A noninvasive neuroimaging approach to facilitate diagnosis or guide surgical planning will ensure better treatment response assessment, ultimately improving patient care^31^.

While MRI remains the most common clinical imaging technique for evaluating CNS tumors^5^, conventional MRI sequences such as T1WI and T2WI correlated poorly with pathologies of high-grade brain tumors. For example, hyperintense regions in T2W and FLAIR images surrounding the Gadolinium (Gd)-enhancing lesion cannot distinguish between infiltrative tumor, vasogenic edema, or immune cell^32^. Gd-enhancement in T1WI also could occur due to either tumor progression or radiation necrosis^33^. Furthermore, conventional T1W and T2W image contrasts vary from scan to scan and are not quantitative, as they depend not only on the MR characteristics of brain tissue, but also on the scanner models, magnet strength, and pulse sequences.

To address the limitations of conventional MRI bridging the gap between histology and MRI for pediatric brain tumor diagnoses, we developed a novel image processing technique, i.e., DHI, taking advantage of previously developed DBSI and DNN algorithm. DBSI provides a simple tensor expression to visualize morphological features resulting from both tumor and non-tumor elements of the brain that are indistinguishable by conventional MRI. In our previous studies, we demonstrated how DBSI-derived restricted fraction positively correlated with adult GBM tumor cellularity identified by H&E staining^17^. In this study, we demonstrated that the hyperintense restricted fraction regions also accurately identify densely cellular tumor areas (Fig. 2). Group analysis across multiple samples with various tumor types also indicated densely cellular tumor had higher restricted fraction values than either normal WM, less densely cellular tumor, infiltrative edges or necrosis. From the areas of necrosis, infiltrating edge, less densely cellular tumor, and densely cellular tumor, we observed a trend towards a gradual increase in restricted fraction values across these four types of histological areas. Thus, the restricted fraction could serve as an appropriate biomarker to assess tumor cellularity in high-grade pediatric brain tumors.

In addition, necrosis exhibited higher values in hindered diffusion fraction and free diffusion fraction than all other histologic components, indicating that these two diffusion metrics are strongly associated with tumor necrosis. Furthermore, our results showed comparable ADC, FA, restricted fraction, and fiber fraction values between the infiltrative edge and normal WM, suggesting that these diffusion metrics lack adequate specificity to distinguish between infiltrating tumor cellularity and white matter. Note that infiltrative edges showed higher isotropic ADC and hindered fraction values than do normal WM, potentially pointing to how tumor infiltration displaces normal parenchyma^34^, destructs of white matter tracts^35^, and/or forming vasogenic edema to disrupt blood brain barrier^36^.

In this study, we demonstrated that DHI, i.e., DBSI-derived metrics as the input classifiers for DNN, differentiates 6 major types of tumor pathologies with an overall accuracy of 85.8%. In detecting and distinguishing individual tumor histology, ROC analysis of DHI models calculated the AUC, sensitivity, and specificity values of all selected tumor pathologies to be higher than 0.94, 88% and 86%, respectively. In the precision-recall analysis, the prediction of infiltrative edge was relatively low for AUC (0.747) and in F_1_-score (0.698), likely due to the highly variable degrees of infiltration or inherent cellularity differences among brain tumors and infiltrated brain regions. For example, infiltrative edges with mild to intermediate tumor cellularity could be falsely predicted to be normal WM. Similar phenomenon was observed from the results of confusion matrix (Fig. 5b).

In contrast to previous studies, we adopted a voxel-wise analysis through precise co-registration between histology and MR images, to bridge MRI and histology. Application of this approach accurately detected distinct regions within pediatric brain tumors that were histologically heterogeneous^37,38^. Image voxels within a region of interest from a specimen could vary differently from each other, reflecting histological heterogeneity. Since DBSI models diffusion-weighted MRI signals independent of neighboring image voxels^10^, each image voxel has its own DBSI metric profiles. Thus, DBSI provides a unique opportunity to assess the heterogeneous tumor pathology-associated structural changes within individual image voxels. DBSI-derived structural metrics are thus ideal to serve as the unique input classifiers for the DNN algorithm. Patient-wise analysis has been typically studied by correlating image metrics with clinical scores or survival rates. There have been attempts to correlate MRI signal with tumor histopathology using stereotactic biopsy^39,40^. However, the analyses have been hindered by spatial misalignment between MRI-defined lesions and biopsy location, in addition to the high histological heterogeneity of high-grade pediatric brain tumors.

Although the relatively small number of subjects (n = 9) limited the broad applicability of the results, we performed voxel-wise analyses (matching the DBSI voxel-based modeling characteristics) on a total of 99,388 image voxels from 45 brain specimens covering different areas of the brain alleviating the limitation on sample size while providing a proof-of-concept demonstration of DHI. The unbalanced data distribution amongst different tumor histologic components imposed another limit since the imbalance could compromise the performance of a DNN model. We have addressed the concern by employing an oversampling approach to balance the training data and adopted precision-recall analyses to provide complement ROC analyses.

In conclusion, we have demonstrated that DHI can accurately characterize and classify multiple histologies in fixed postmortem specimens of pediatric high-grade brain tumors. While precise prediction of infiltrative edges was suboptimal, the collective findings are encouraging. The efficacy of DHI classification of pediatric brain tumor pathologies still requires in vivo application with image-guided stereotactic biopsy validation.

## Data Availability

The datasets generated during and/or analyzed during the current study are not publicly available due to sensitive patient information but are available from the corresponding author on reasonable request.
